# A Longitudinal Assessment of Metabolic Syndrome

**DOI:** 10.3390/jcm14030747

**Published:** 2025-01-24

**Authors:** Dilbar Aidarbekova, Karlygash Sadykova, Yerbolat Saruarov, Nursultan Nurdinov, Mira Zhunissova, Kumissay Babayeva, Dinara Nemetova, Ainur Turmanbayeva, Aigerim Bekenova, Gulnaz Nuskabayeva, Antonio Sarria-Santamera

**Affiliations:** 1 Department of Special Clinical Disciplines, Faculty of Medicine, Khoja Akhmet Yassawi International Kazakh-Turkish University, Turkistan 161200, Kazakhstan; 2 Department of Fundamental Sciences, Faculty of Medicine, Khoja Akhmet Yassawi International Kazakh-Turkish University, Turkistan 161200, Kazakhstan; yerbolat.saruarov@ayu.edu.kz; 3 Department of Fundamental Medical Sciences, Faculty of Dentistry, Khoja Akhmet Yassawi International Kazakh-Turkish University, Turkistan 161200, Kazakhstan; 4 Department of Biomedical Sciences, School of Medicine, Nazarbayev University, Astana 010000, Kazakhstan

**Keywords:** adult, cohort studies, metabolic syndrome, epidemiology, risk factors

## Abstract

**Background/Objectives**: Metabolic syndrome (MetS) is a combination of conditions including central obesity, high blood pressure, high glucose levels, and abnormal triglycerides and cholesterol, which together increase the chances of heart disease, diabetes, and even death. The rates of MetS are different around the world, with 20–30% in Europe and 21.8–23.9% in Kazakhstan. Because MetS changes over time, it is important to study the dynamics of their components to improve prevention and treatments. This work aims to obtain the incidence of MetS and to evaluate the specific components associated with the emergence of new MetS cases in this population. **Methods**: This is a longitudinal study with a 10-year follow-up in Turkestan city between 2012 and 2024. Information was collected through physical exams, blood tests, and anthropometric measurements. Logistic regression and ROC curve analysis were used to find which factors increase the incidence of MetS. **Results**: Among 434 participants analyzed (78.8% women, and mean age 40.87, 66% < 2 MetS components, 20% had MetS blood pressure, and 65.9% had MetS waist circumference), the incidence of MetS at follow-up was 40%. The key risk factors for newly diagnosed MetS included elevated blood pressure and increased waist circumference. Multivariate analyses highlighted these components as the strongest predictors of MetS, with significant associations observed for participants with two or more MetS components at baseline. **Conclusions**: Elevated blood pressure and central obesity were identified as pivotal contributors to MetS progression. Given the rising prevalence of Mets and its implications, these results show the need to start treatment and check these risks early to prevent serious health problems.

## 1. Introduction

Metabolic syndrome (MetS) is a combination of conditions, including central obesity, high blood pressure, elevated blood glucose, and abnormal cholesterol levels, that together raise the risk of cardiovascular diseases (CVD), type 2 diabetes (T2DM), and mortality from any cause [1]. The dynamics of MetS are driven by multiple factors, including genetic background, lifestyle choices, and metabolic issues [2]. This syndrome has become a major global health concern due to its increasing prevalence [3] and its connection to serious health problems [4].

MetS is associated with an increased risk for the development of type 2 diabetes mellitus and cardiovascular disease. People with the syndrome are twice as likely to die from a macrovascular event and three times as likely to have ischemic heart disease and stroke compared with people without the MetS. This syndrome is affecting the general population in epidemic proportions and is frequently associated with increased risk of cardiovascular morbidity and mortality [5].

The growing prevalence of non-communicable diseases is a major global public health concern. This significant burden is in part due to the global increase in the constellation of metabolic dysregulation processes affecting obesity, glucose homeostasis, lipid metabolism, and elevated blood pressure [6]. Many of these diseases occur associated, share common risk factors, and are linked with increased risks of premature deaths [7].

Cardiovascular diseases represent the leading cause of premature mortality in Central Asian countries [8,9]. In these countries, there is significantly low awareness of risk factors [10] as well as a low level of their detection by the healthcare system [11].

The prevalence of MetS in most countries of the European World Health Organization (WHO) region varies between 20 and 30%, with relatively equal distribution across genders [12,13]. Previous analyses in Kazakhstan have reported a prevalence of MetS among women of 21.8% and 23.9% for men, based on IDF criteria. Abdominal obesity was the most prevalent component of MetS, followed by hyperglycemia, and systolic and diastolic hypertension [14]. One problem that complicates assessing the epidemiology of MetS and for comparison between countries and settings is the use of different definitions by different researchers.

The evolution of MetS and its distinct components is not linear, involving shifts between different levels of metabolic health, from mild to severe disorders. Knowing the factors associated with these changes is crucial for making effective treatments that can stop MetS from worsening and, ideally, reverse some of its effects [15]. Multi-state models, such as the Markov model are used to predict these changes and give insights into both the worsening and improvement of MetS components [16].

There is no evidence of the progression of the constellation of dysregulations associated with MetS in Kazakhstan. The specific cultural, geographic, healthcare, and even genetic characteristics of the Kazakh population suggest the relevance of exploring MetS in this population.

In this research, we analyze the progression to MetS in a general adult population in Kazakhstan. Examining the main mechanisms and contributing factors, this study aims to obtain the incidence of MetS and to evaluate the specific components associated with the emergence of new MetS cases in this population, improving our understanding of how MetS progresses, which in future can help to create more effective prevention and treatment strategies.

## 2. Materials and Methods

This is a longitudinal study that involved the collection of data from 938 participants initially recruited between 2012 and 2014 residing in Turkestan city and who were invited for a second interview and blood tests in 2024. Patients were recruited from the Khoja Akhmet Yassawi International Kazakh-Turkish University Outpatient Clinic in Turkestan.

### 2.1. Ethical Approval and Informed Consent

After the ethical committee approval, participants were thoroughly informed about the study’s objectives, methodology, potential risks, and anticipated benefits. Detailed explanations were provided to ensure that participants fully understood each aspect of the study, including the procedures, their roles, and any associated commitments. Following this comprehensive briefing, informed consent was obtained from all participants, confirming their voluntary agreement to participate. This consent process adhered to ethical standards and was documented to confirm that each patient had the opportunity to ask questions and had received satisfactory answers before consenting.

### 2.2. Study Design

Participants of the study were reviewed through the national medical database and contacted by phone with the help of their general practitioners.

After informed consent, participants underwent a physical examination, blood draw, and questionnaire. Anthropometric measurements: height, weight, neck, waist, and hip measurements are taken. Height was measured by a stadiometer (Medtechnika, Tula, Russia), in which the study participants stood straight, without outerwear and shoes, and heels, buttocks, and shoulders were in contact with the vertical plane of the stadiometer. The patients’ heads were kept in the “Frankfurt plane” where the lower boundaries of the orbits were in the same horizontal plane as the external auditory space. When holding their breath on inspiration, the stadiometer plate was lowered to the head of the patient, after which the subject departed. After taking three measurements, the average growth index was determined with an accuracy of 1 cm. Body weight was measured on an electronic Detecto scale (Webb City, MO, USA). After turning on the scale display to check the performance, when 0.00 g appeared, the participants were asked to stand on the scale. At the same time, shoes, outerwear, and heavy items in pockets (mobile phones, wallets, etc.) were removed. Study participants stood in the center of the scales with their arms freely at their sides. At the same time, the patients looked straight and remained motionless. After three measurements, the mean body weight was recorded to the nearest 0.1 kg. Based on the results of measuring height and body weight, the BMI was determined by the following formula: weight (kg)/height in m^2^. Waist circumference (WC) was measured while standing, using a soft centimeter tape with an accuracy of 0.1 cm. WC was measured after normal expiration in the middle between the lower rib and the upper part of the iliac crest.

Glucose levels were measured with a glucometer (ATCare, Peristeri, Greece) using capillary blood from the fingertip, both fasting and two hours after a standardized breakfast. A complete blood count was conducted on a BS-6000 Mindray device (Shenzhen, China), with 3 mL of blood collected in a purple-top EDTA tube. HOMA-IR and HOMA-β were calculated and divided into tertiles. HOMA models were calculated as HOMA-IR = fasting insulin (lU/mL) × fasting glucose (mmol/L)]/22.5, and HOMA-β = [20 × fasting insulin (lU/mL)]/[fasting glucose (mmol/L) − 3.5]. Impaired Fasting Glucose (IFG) was defined as a fasting plasma glucose of 6.1–6.9 mmol/L.

For this study, we used the definition of MetS according to the 2006 International Diabetes Federation (IDF) criteria, and MetS is diagnosed when three or more criteria are met: triglycerides of 150 mg/dL or greater; high-density lipoproteins (HDL-cholesterol) < 40 mg/dL in men and <50 mg/dL in women; BP 130/85 mmHg or greater; fasting glucose of 100 mg/dL or greater; waist circumference > 90 cm in men and >80 cm in women [17].

Exclusion criteria were as follows: cases meeting the criteria for MetS in 2014, patients lost to follow-up, moved to other locations, and passed away.

### 2.3. Statistical Methods

Chi-square tests and mean comparisons were performed, followed by multivariable logistic regression. Three multivariable logistic models were analyzed using diverse types of variables: (1) considering the values of the different parameters to estimate MetS; (2) considering the components that define MetS; and (3) the number of components to be considered to define MetS. To assess the predictive capacity of the logistic regression models, receiver operating characteristic (ROC) curve analysis was conducted.

## 3. Results

**Figure 1:**
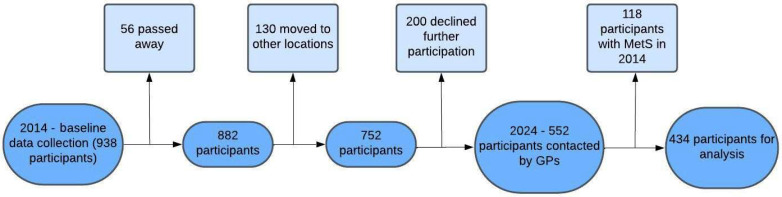
Flow chart of the studied population.

Of the 938 participants in the baseline data collection in 2012 to 2014, 56 participants have since passed away. It was not possible to contact 130 of them, probably due to changes in residence to other locations, and 200 declined further participation. The remaining 552 participants were analyzed. Figure 1 depicts the flow chart of the data.

Out of 552 participants, 21.2% were represented by males and 78.8% by females with a mean age of 40.9 at baseline and 52.9 at follow-up. The incidence of newly diagnosed MetS was 40.3%.

Table 1 represents the main characteristics of the population in the two periods of time analyzed, baseline and follow-up. The prevalence of the two more frequent components of MetS (blood pressure and waist circumference) increased from 20.5% and 65.9%, to 33.9% and 74.4%.

**Figure 2:**
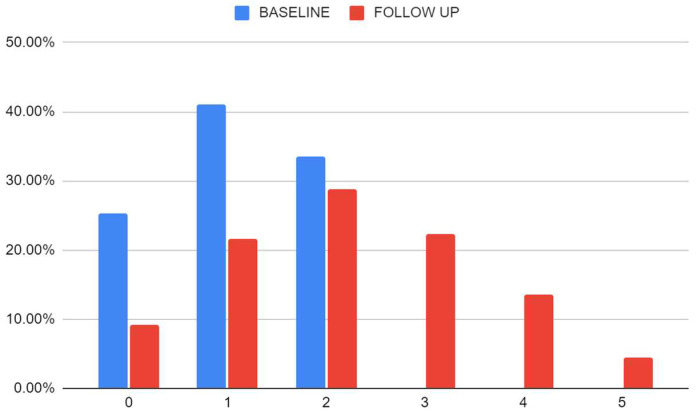
Distribution of participants at baseline and follow-up by the number of metabolic syndrome components.

The values of all the MetS components analyzed showed a worsening in the follow-up. To highlight that at baseline, 66% had <2 MetS components, while at follow-up only 31%. The number of cases that met the criteria for MetS at follow-up (Figure 2) was 40.3%. Almost 15% had four or more components at follow-up. In Supplementary Table S1, we can see the frequency of aggregation of the different MetS components at baseline.

**Figure 3:**
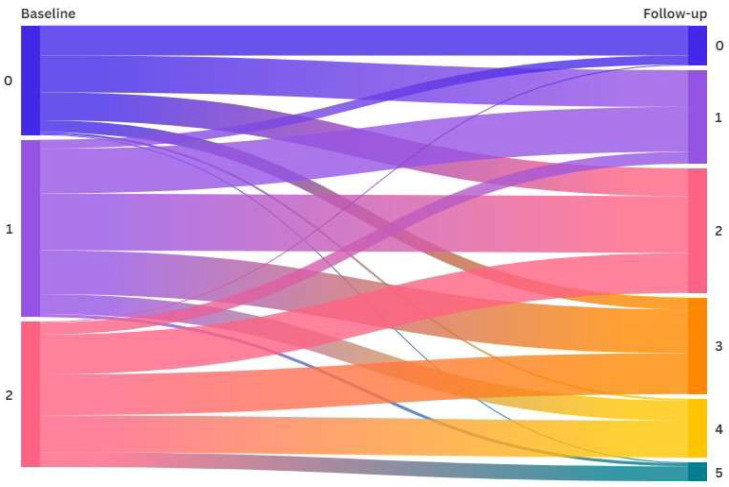
Sankey diagram reflecting the transition of participants by the number of metabolic syndrome components at baseline and follow-up.

Some participants showed a deterioration of their MetS components between baseline and follow-up. The increase in the number of components was especially relevant for those with two MetS components at baseline. Most cases deteriorated the number of MetS components from baseline to follow-up (68.4%). Only 26.5% maintained the number of MetS components, and 5.1% had a lower number of components (Figure 3).

Table 2 reports the risk associated with the incidence of MetS by age and sex, by the number of components, and by the presence of specific components at baseline. Age was associated with a higher incidence, but not sex. The results showed a significant association of the incidence of MetS with the increase in the number of MetS at baseline, as well as with two of the MetS components: elevated blood pressure and higher waist circumference values.

Table 3 indicates that all parameters were higher at baseline for participants that developed MetS at follow-up, including age, except for HDL, and that worsening of all, except for HDL and glucose, were associated with a higher risk of developing MetS at follow-up.

**Figure 4:**
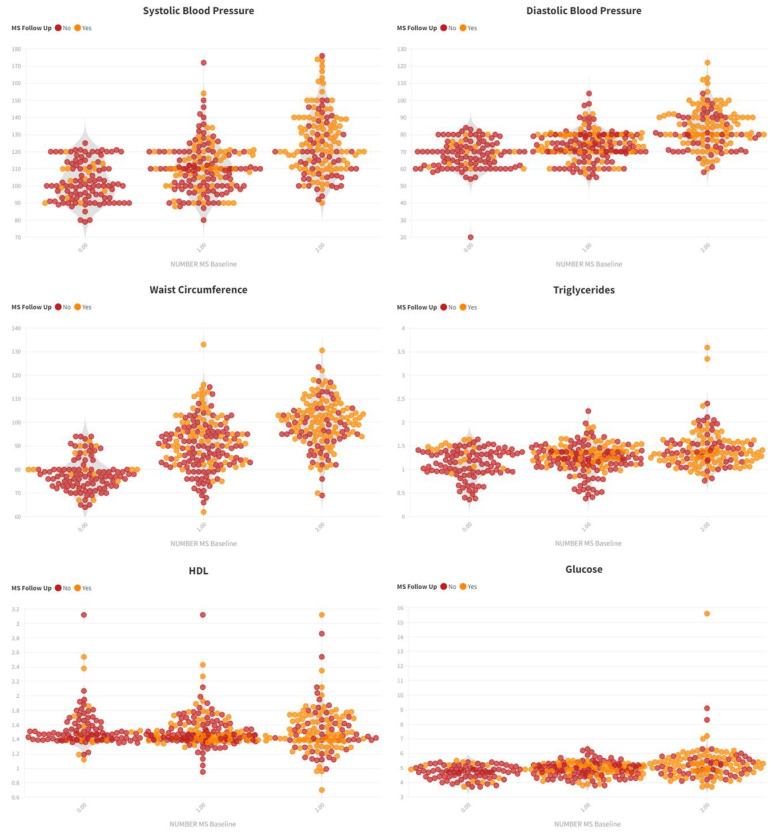
Violin graphs representing the incidence of metabolic syndrome at follow-up based on metabolic syndrome components, and the number of affected metabolic components at baseline.

In Figure 4, it could be observed that elevated blood pressure and waist circumference were highly prevalent in cases with two MetS components and that those factors were strongly associated with new cases of MetS at follow-up.

**Figure 5:**
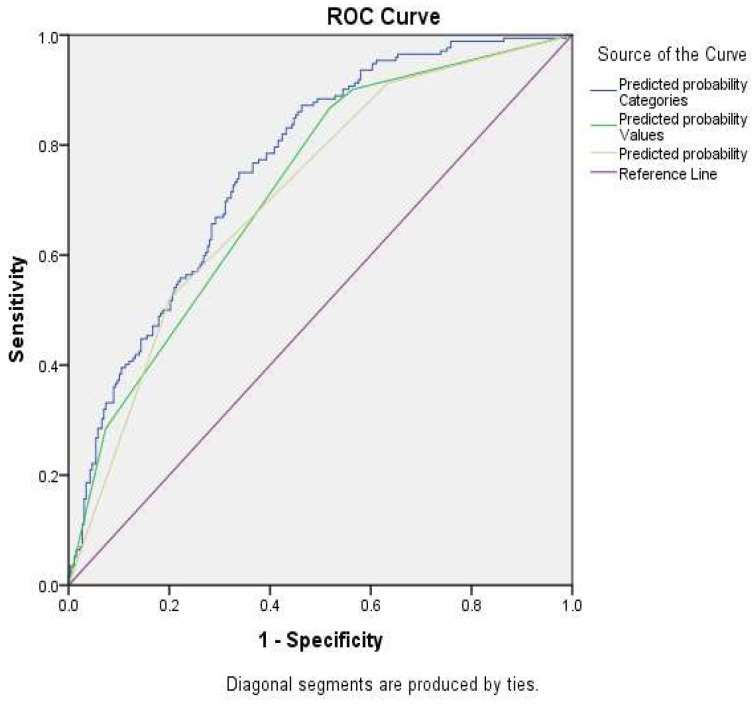
Area under the curve of the 3 multivariable models to calculate their discrimination predictive capacity.

Table 4 shows the results of the three multivariate logistic regression analyses with different exposure variables, and Figure 5, the AUC results of the models. The model including the MetS components raised blood pressure and raised waist circumference is the model with the highest predictive value as estimated by AUC (Figure 5). The AUC were, respectively, as follows: components of MetS, 0.76; values of MetS components, 0.72; and for the number of MetS components, 0.71.

This model includes the MetS components blood pressure and MetS WC, which are the two characteristics that were independently associated with a higher risk of newly diagnosed MetS. Although age showed an association in univariate analysis, its effect did not remain significant in multivariate analyses. No differences between men and women were identified in these analyses.

In Table 5, we can see the lack of association between IFG, IGT, insulin resistance, and beta cell deficit with the incidence of MetS.

## 4. Discussion

The main finding of this analysis that explored the progression of MetS and its components in a general adult population is an incidence of 40% of MetS after 10 years of follow-up. The second is that the most relevant factors that increased the risk of newly diagnosed MetS in the follow-up period are elevated blood pressure and waist circumference. Both factors, blood pressure and waist circumference, were independently associated in multivariable models that indicated a reasonable capacity to predict the 10-year incidence of MetS in this population. Another finding of this study is that, although blood pressure and waist circumference are strongly associated, the higher number of MetS components affected also increases the risk of incidence of MetS. The incidence of new cases of MetS was found to be higher in those with already two MetS components at baseline, but it also was found in cases with one or even zero components. A relevant finding of this study is the lack of association between glucose levels or glucose metabolism indexes, including insulin resistance, and the incidence of MetS in this population.

MetS is a complex disorder with multiple factors that can interchange with time. Although numerous studies have aimed to determine the pathogenesis of this condition, the exact pathways linked with the development of MetS are still unclear due to the complexity of the disease and the many factors that contribute to it. Factors that have been described as pivotal in the incidence and progression of MetS are low-grade inflammation driven by excess visceral fat, which disrupts glucose metabolism and increases CVD and T2DM risks [18], as well as insulin resistance [19] and lipid metabolism dysregulation [20]. The influence of genetic predisposition is also being increasingly recognized [21,22].

Elevated blood pressure (BP) is a significant component of MetS and one of the criteria for this diagnosis. Elevated BP levels are associated typically with the incidence of MetS and are considered a strong precursor of MetS, highlighting the importance of early BP management to prevent the syndrome’s onset. A five-year retrospective cohort study examined the relationship between BP levels and the incidence of MetS. The study found that individuals with higher baseline BP were more likely to develop MetS over time [23], while people with optimal and normal BP levels were less susceptible to developing MetS over time, suggesting that abnormal BP could be considered as a pre-existing phase of MetS and the relevance for maintaining normal BP for MetS prevention [24,25].

The pathophysiology that connects high BP to MetS is explained by several interconnected mechanisms. Central obesity and abnormal levels of lipids may cause endothelial dysfunction, reducing the availability of nitric oxide and affecting proper vasodilation, which is an important factor in how MetS develops. Inflammation, which is at a low level but constant in both conditions, makes this worse by triggering more inflammatory reactions that encourage the progression of MetS. Understanding how BP contributes to MetS shows how important it is to check CVD risks properly and take timely actions. If high BP is managed early, it might help stop or delay MetS and its complications [26].

Waist circumference (WC) is a crucial marker for central obesity and a significant contributor to MetS. Central obesity is strongly associated with insulin resistance, dyslipidemia, and hypertension—key components of MetS. The rising prevalence of obesity is identified as a primary driver of MetS, emphasizing prevention and management strategies. A comprehensive review of the connection between obesity, MetS, and T2DM has stressed that these conditions have similar mechanisms and often happen one after another, increasing the risk of CVD events. The rising number of obesity cases is pointed out as a main cause of MetS, showing the importance of good prevention and management plans [27]. Dysfunctional adipose tissue in obesity contributes to insulin resistance and lipid metabolism disorders, both central to MetS development [28].

WC has also been identified as a valid estimate for predicting changes in body composition related to MetS. In the dynamic behavior of MetS progression, central obesity, as indicated by increased WC, often precedes other MetS components. Regularly measuring WC is an effective way to identify individuals at risk for MetS [29]. Targeted lifestyle interventions focused on weight reduction are essential to prevent or delay the onset of MetS. In clinical practice, WC measurement is a simple and cost-effective method for assessing central obesity and evaluating MetS risk [30]. Current guidelines provide population-specific WC thresholds to identify those at higher risk, ensuring tailored preventive strategies [31].

Although insulin resistance is considered a major pathogenic factor playing a crucial role in the development of MetS and has been proposed as a hallmark and one of its critical underlying causes [32,33], in our study, glucose metabolism indexes—including both insulin resistance and pancreatic dysfunction—did not show a significant association with an increased risk of MetS incidence. During the early stages of insulin resistance, elevated insulin secretion may temporarily compensate to maintain metabolic balance, potentially masking its relationship with MetS. Other factors, such as inflammation, oxidative stress, or genetic predispositions, may independently drive MetS, diminishing the apparent role of insulin resistance. Since MetS is a cluster of risk factors—including obesity, dyslipidemia, hypertension, and hyperglycemia—individuals may meet its diagnostic criteria through combinations less dependent on insulin resistance, such as hypertension influenced by non-metabolic factors. In this population, waist circumference may reflect a predominance of subcutaneous fat rather than visceral fat, which is more metabolically active. As insulin resistance is more strongly linked to visceral fat, variations in fat distribution could obscure this relationship. Additionally, variability in metabolic factors, fat distribution, and population-specific characteristics, such as the unique ethnic traits of the Kazakh population, may further explain the lack of association observed in our study.

MetS is a significant public health concern in Kazakhstan, contributing to the high burden associated with the high incidence and mortality of CVDs in the country [34]. Several studies have explored the prevalence, pathogenesis, and clinical features of MetS within the Kazakh population. A 2018 study reported a prevalence of MetS among civil servants aged 35–70 of 40.3%, compared to 32.8% in the general population of the same age group. Both men and women across different age categories showed a higher incidence of individual symptoms and the syndrome compared to population averages. Specifically, the rates of arterial hypertension, hypercholesterolemia, and carbohydrate metabolism disorders were significantly higher [35]. A study conducted among the Kazakh population in the Xinjiang region of China examined the relationship between MetS and the development of CVD [32]. The cohort study lasted for about 5.49 years and included 2644 participants. The results showed that elevated blood pressure, increased waist circumference, and high triglyceride levels were independent risk factors for CVD. Participants with multiple components of MetS had an increased risk of developing CVD, indicating the cumulative effect of these risk factors. Another study assessing the prevalence of MetS in southern Kazakhstan found that abdominal obesity was the most prevalent component of MetS in women (74.3%), followed by hyperglycemia (26.5%) and diastolic hypertension (25.5%), while the most common components for men were abdominal obesity (70.7%), systolic hypertension (44.4%), and diastolic hypertension (40.0%) [36].

This study has several limitations. First, the relatively small sample size reduced the statistical power of the analysis. Additionally, imbalances in gender, with a sample composed of almost 80% of women, and age distribution, with a mean age of 52 at follow-up, posed challenges for conducting robust subgroup analyses and may have introduced bias into the results. Variations in the definition of MetS may be associated with differences in results identified in other studies [37]. This study does not address the interrelationships between the different MetS components and how these interactions may increase the risk of their progression [38]. This study has not analyzed either the possible effects of socioeconomic variables on MetS dynamics [39] or lifestyle factors, physical activity, diet, smoking, alcohol consumption, or the impact of any medical care interventions, pharmacological or not pharmacological [40]. Inflammatory markers were not included in these analyses [41]. Turkestan population characteristics, mostly composed of ethnic Kazakhs and Uzbeks, and their genetic characteristics may also contribute to explaining the specific findings of this work, but also potentially limit their generalizability [42]. In this study, capillary glucose was measured at baseline. Glucose meters designed for capillary blood demonstrate acceptable accuracy when compared with venous samples in healthy individuals. Still, greater variation is observed in critically ill patients with impaired perfusion [43]. Capillary samples may result in inaccurate glucose measurements in critically ill patients, particularly those in shock [44]. Except in those patients, capillary blood could be an alternative for venous blood glucose measurements [45,46].

This study also has relevant strengths. This is a prospective study with a more than 10-year follow-up conducted in the general population in Kazakhstan. Typical studies investigate the prevalence of MetS and its components, but there are limited data about MetS profiles and their incidence not just in the Central Asian population but globally [47]. This study provides important insights into its dynamic progression and the specific risks associated with its components.

The evidence of the factors that represent a risk for MetS are well known: lifestyle habits, including physical inactivity, eating an unhealthy diet, not obtaining enough good-quality sleep, smoking, and excessive alcohol drinking [48]. Other factors that have also been considered are age, family history, and genetics, as well as low socioeconomic status and the coexistence of other medical conditions. The characteristics of the data collected at two moments separated by more than 10 years, prevent us from establishing which factors are specifically associated with the incidence of MetS in this population. However, it may be estimated that the above-mentioned factors could be linked to the significant incidence of MetS observed in our study.

These factors have been associated with incidence studies obtained in a variety of countries. Data from the US reported an incidence of 33.8% in the general population, with a worrying significant upward trend over the years [49]. In Asia–Pacific countries, secular trends show an increase in prevalence of about 50–75% over 10 years, highlighting the need for newer well-designed epidemiological studies to identify the most recent extent of the MetS epidemic [50]. A study in India reported an incidence of 32.1 per 1000 persons/year [51], and the incidence in Iran was estimated to be 5.45% [52].

Data on the epidemiological situation of MetS of Kazakhstan and Central Asia has not been previously reported. Central Asian populations exhibit unique genetic markers that may differ from those found in East Asians or Europeans [53]. Located in the Eurasian heartland, Central Asia has been a crossroads for migrations, invasions, and commercial routes (Silk Road). The intricate history may have shaped patterns of genetic variability in a complex manner and may have led to varied susceptibilities to MetS components. The specific geographic and climatological conditions of the zone have also significantly influenced the dietary habits of Central Asian populations, which were traditionally based on high levels of animal fats, red meat, and refined carbohydrates, although in recent years, urbanization has increased the consumption of processed and high-calorie foods, exacerbating MetS risks [54].

The main implication of this study is that without a focused approach to the control of the constellation of the dysregulations associated with MetS, there is a natural tendency to increase. In 10 years, 70% of participants showed an increase in any of the MetS components, and 40% of them had three or more. High blood pressure, dietary risks, tobacco, high body-mass index, high alcohol use, and high fasting plasma glucose represent the most frequent factors for death and disability in Kazakhstan [54], a country with high cardiovascular mortality. Without targeted interventions and strengthening preventive and health promotion measures, pharmacological and non-pharmacological, focusing on lifestyle modifications, including improvements in physical activity and diet, these results suggest that the reduction in the high burden of morbi-mortality attributed to MetS may be complex to alleviate in Kazakhstan [55]. This is a worrying finding since Kazakhstan is experiencing a significant increase in its older population, which may cause the prevalence of MetS and the attributable cardiovascular effects to increase even more than it already has.

## 5. Conclusions

To the best of our knowledge, this longitudinal study is the first study conducted in the Kazakh adult population that provides valid estimates of the incidence of MetS and its components, suggesting targets that public health and clinical interventions would have to focus on to curb the MetS pandemic in Kazakhstan. Logistic regression models and ROC analysis determined that the strongest predictors of MetS development are high blood pressure and increased waist circumference. Models including these parameters showed high prediction accuracy. Despite the association of age with an increased risk of MetS, this dependence was insignificant in multivariate analysis. No effect was found related to insulin resistance. This study highlights the variable nature of MetS and emphasizes the importance of timely diagnosis and strengthening health promotion and preventive measures to reduce its prevalence both in Kazakhstan and in other regions to alleviate the already high burden of morbi-mortality associated with cardiovascular diseases in the country.

## Figures and Tables

**Table 1:** Main characteristics of the studied population at baseline and during follow-up.

Categories		Baseline	Follow-Up	*p* Values
Sex	Male	21.2%		
	Female	78.8%		
Age Mean (SD)		40.9 (10.9)	52.9 (10.7)	
	<36 years	34.3%	5.7%	
	36–45 years	29.7%	22.5%	
	>45 years	35.9%	71.8%	
Systolic Blood Pressure Mean (SD); Median		113.29 (17.19); 110	120.37 (18.56); 120	<0.001 *
Diastolic Blood Pressure Mean (SD); Median		75.42 (11.28); 75	78.25 (11.17); 80	<0.001 *
Waist Circumference Mean (SD); Median		90.169 (14.17); 90	93.90 (14.20); 94	<0.001 *
Triglycerides Mean (SD); Median		1.28 (0.35) 1.29	1.55 (0.71); 1.37	<0.001 *
HDL Mean (SD); Median		1.52 (0.27); 1.46	1.34 (0.61); 1.23	<0.001 *
Glucose Mean (SD); Median		4.98 (0.84); 4.9	5.29 (1.22); 5.4	<0.001 *
Raised Blood Pressure		20.5%	33.9%	<0.001 **
Raised Glucose		10.8%	26.5%	0.001 **
Raised Triglycerides		6.5%	36.9%	0.497 **
Raised Waist Circumference		65.9%	74.4%	<0.001 **
Reduced HDL		4.6%	50.9%	0.027
Number of MetS Components Mean (SD)		1.08 (0.76)	2.23 (1.30)	<0.001
Number of MetS Components Median		1	2	
Metabolic Syndrome		–	40.3%	
Total		434		

MetS: metabolic syndrome; SD: Standard deviation. * Wilcoxon ranked test ** Chi-square test.

**Table 2:** Incidence of metabolic syndrome in follow-up by age, sex and metabolic syndrome components at baseline.

	Incidence of Metabolic Syndrome%	*p*-Value
<36 years	26.8%	<0.001
36–45 years	43.4%	
>45 years	50.6%	
Men	44.6%	0.350
Women	39.2%	
0 MetS Component	13.6%	<0.001
1 MetS Component	37.6%	
2 MetS Components	63.7%	
Raised Blood Pressure	64.0%	<0.001
Raised Glucose	51.1%	0.112
Raised Waist Circumference	53.15	<0.001
Raised Triglycerides	50.0%	0.280
Reduced HDL	40.8%	0.335

**Table 3:** Means values of metabolic syndrome components at baseline and incidence of metabolic syndrome in 2024.

	Age	SBP	DBP	WC	TG	HDL	Glucose
No MetS at follow-up	38.69	108.36	72.39	85.710	1.21	1.51	4.83
MetS at follow-up	44.09	118.66	79.06	96.769	1.37	1.52	5.05
*p*-value	<0.001	<0.001	<0.001	<0.001	<0.001	0.631	0.015
OR incidence of MetS IC95%	1.05 1.0–1.07	1.04 1.02–1.05	1.06 1.04–1.08	1.08 1.06–1.10	4.29 2.24–8.23	1.20 0.58–2.47	1.35 0.99–1.84

**Table 4:** Multivariable logistic regression of incidence of metabolic syndrome: (A) by values of components of metabolic syndrome at baseline. (B) by components of metabolic syndrome at baseline. (C) by number of metabolic syndrome components at baseline.

**(A)**	**OR**	**IC95%**
Diastolic Blood Pressure	1.04	1.02–1.07
Waist Circumference	1.06	1.04–1.08
**(B)**	**OR**	**IC95%**
Raised Blood Pressure	2.97	1.77–4.99
Raised Waist Circumference	5.71	3.43–9.51
**(C)**	**OR**	**IC95%**
0 MetS Components	REF	
1 MetS Components	3.82	2.05–7.13
2 MetS Components	11.11	5.86–21.09

**Table 5:** Metabolic syndrome incidence based on glucose metabolic components at baseline.

		Categories	Incidence of MetS	*p* Values
HOMA IR	Mean 1.71		OR 1.06 (0.75–1.48)	
	Median 1.63			
Tertiles of IR		42.6%	36.0%	*p* = 0.989
		44.5%	35.5%	
		12.9%	37.0%	
HOMA beta	Mean 169.46		OR 1.00 (0.99–1.00)	
	Median 115.14			
Tertiles of beta cell deficit		5.7%	25.0%	*p* = 0.678
		14.3%	33.3%	
		80.0%	36.9%	
IFG		2.4%	OR 2.38 (0.76–7.43)	

## Data Availability

Data can be obtained from the corresponding author upon request.

## Supplementary Materials

The following supporting information can be downloaded at: https://www.mdpi.com/article/10.3390/jcm14030747/s1, Table S1. Frequency of the combination of metabolic syndrome components at baseline (in %).
