# Supplementary material for: A Longitudinal Assessment of Metabolic Syndrome
Source: J Clin Med. 2025 Jan 24;14(3):747. doi: 10.3390/jcm14030747 (PMC11818716; doi:10.3390/jcm14030747)
Supplement: Supplementary file 1 [file jcm-14-00747-s001.zip › jcm-3398899-supplementary.pdf]

Supplementary Table S1. Frequency of the combination of metabolic syndrome components at baseline (in %).

	Blood Pressure	Glucose	Triglycerides	Waist Circumference	HDL
Blood Pressure		1.12	3.37	79.78	0.00
Glucose	2.13		0.00	76.60	2.13
Triglycerides	10.71	0.00		64.29	3.57
Waist Circumference	24.83	12.59	6.29		5.24
HDL	0.00	5.00	5.00	75.00	
